# The co-solvent Cremophor EL limits absorption of orally administered paclitaxel in cancer patients

**DOI:** 10.1054/bjoc.2001.2118

**Published:** 2001-11

**Authors:** M M Malingré, J H M Schellens, O Van Tellingen, M Ouwehand, H A Bardelmeijer, H Rosing, F J Koopman, M E Schot, W W Ten Bokkel Huinink, J H Beijnen

**Affiliations:** Department of Medical Oncology, The Netherlands Cancer Institute/Antoni van Leeuwenhoek Hospital, Plesmanlaan 121, Amsterdam, 1066, The Netherlands; Department of Pharmacy and Pharmacology, The Netherlands Cancer Institute/Slotervaart Hospital, Louwesweg 6, Amsterdam, 1066, The Netherlands; Division of Drug Toxicology, Faculty of Pharmacy, Utrecht University, Sorbonnelaan 16, Utrecht, CA, 3584, The Netherlands and; Department of Clinical Chemistry, The Netherlands Cancer Institute/Antoni van Leeuwenhoek Hospital, Plesmanlaan 121, Amsterdam, CX, 1066, The Netherlands

**Keywords:** paclitaxel, oral administration, Cremophor EL

## Abstract

The purpose of this study was to investigate the effect of the co-solvents Cremophor EL and polysorbate 80 on the absorption of orally administered paclitaxel. 6 patients received in a randomized setting, one week apart oral paclitaxel 60 mg m^−2^ dissolved in polysorbate 80 or Cremophor EL. For 3 patients the amount of Cremophor EL was 5 ml m^−2^, for the other three 15 ml m^−2^. Prior to paclitaxel administration patients received 15 mg kg^−1^ oral cyclosporin A to enhance the oral absorption of the drug. Paclitaxel formulated in polysorbate 80 resulted in a significant increase in the maximal concentration (C _max_) and area under the concentration–time curve (AUC) of paclitaxel in comparison with the Cremophor EL formulations (*P* = 0.046 for both parameters). When formulated in Cremophor EL 15 ml m^−2^, paclitaxel C _max_ and AUC values were 0.10 ± 0.06 μM and 1.29 ± 0.99 μM h^−1^, respectively, whereas these values were 0.31 ± 0.06 μM and 2.61 ± 1.54 μM h^−1^, respectively, when formulated in polysorbate 80. Faecal data revealed a decrease in excretion of unchanged paclitaxel for the polysorbate 80 formulation compared to the Cremophor EL formulations. The amount of paclitaxel excreted in faeces was significantly correlated with the amount of Cremophor EL excreted in faeces (*P* = 0.019). When formulated in Cremophor EL 15 ml m^−2^, paclitaxel excretion in faeces was 38.8 ± 13.0% of the administered dose, whereas this value was 18.3 ±15.5% for the polysorbate 80 formulation. The results show that the co-solvent Cremophor EL is an important factor limiting the absorption of orally administered paclitaxel from the intestinal lumen. They highlight the need for designing a better drug formulation in order to increase the usefulness of the oral route of paclitaxel © 2001 Cancer Research Campaign   http://www.bjcancer.com


*British Journal of Cancer* (2001) **85**(10), 1472–1477

© 2001 Cancer Research Campaign doi: 10.1054/ bjoc.2001.2118, available online at http://www.idealibrary.com on IDEAL

http://www.bjcancer.com

### The co-solvent Cremophor EL limits absorption of orally administered paclitaxel in cancer patients

**MM Malingré[1,2], JHM Schellens[1,3], O Van Tellingen[4], M Ouwehand[4], HA Bardelmeijer[4], H Rosing[2], FJ Koopman[2], SE Jansen[2], ME Schot[1], WW Ten Bokkel Huinink[1] and JH Beijnen[1,2,3]**

[1]Department of Medical Oncology, The Netherlands Cancer Institute/Antoni van Leeuwenhoek Hospital, Plesmanlaan 121, 1066 CX Amsterdam, The Netherlands; [2]Department of Pharmacy and Pharmacology, The Netherlands Cancer Institute/Slotervaart Hospital, Louwesweg 6, 1066 EC Amsterdam, The Netherlands; [3]Division of Drug Toxicology, Faculty of Pharmacy, Utrecht University, Sorbonnelaan 16, 3584 CA Utrecht, The Netherlands and [4]Department of Clinical Chemistry, The Netherlands Cancer Institute/Antoni van Leeuwenhoek Hospital, Plesmanlaan 121, 1066 CX Amsterdam, The Netherlands

**Summary** The purpose of this study was to investigate the effect of the co-solvents Cremophor EL and polysorbate 80 on the absorption of orally administered paclitaxel. 6 patients received in a randomized setting, one week apart oral paclitaxel 60 mg m$^{-2}$ dissolved in polysorbate 80 or Cremophor EL. For 3 patients the amount of Cremophor EL was 5 ml m$^{-2}$, for the other three 15 ml m$^{-2}$. Prior to paclitaxel administration patients received 15 mg kg$^{-1}$ oral cyclosporin A to enhance the oral absorption of the drug. Paclitaxel formulated in polysorbate 80 resulted in a significant increase in the maximal concentration ($C_{max}$) and area under the concentration–time curve (AUC) of paclitaxel in comparison with the Cremophor EL formulations ($P = 0.046$ for both parameters). When formulated in Cremophor EL 15 ml m$^{-2}$, paclitaxel $C_{max}$ and AUC values were 0.10 ± 0.06 μM and 1.29 ± 0.99 μM h$^{-1}$, respectively, whereas these values were 0.31 ± 0.06 μM and 2.61 ± 1.54 μM h$^{-1}$, respectively, when formulated in polysorbate 80. Faecal data revealed a decrease in excretion of unchanged paclitaxel for the polysorbate 80 formulation compared to the Cremophor EL formulations. The amount of paclitaxel excreted in faeces was significantly correlated with the amount of Cremophor EL excreted in faeces ($P = 0.019$). When formulated in Cremophor EL 15 ml m$^{-2}$, paclitaxel excretion in faeces was 38.8 ± 13.0% of the administered dose, whereas this value was 18.3 ±15.5% for the polysorbate 80 formulation. The results show that the co-solvent Cremophor EL is an important factor limiting the absorption of orally administered paclitaxel from the intestinal lumen. They highlight the need for designing a better drug formulation in order to increase the usefulness of the oral route of paclitaxel © 2001 Cancer Research Campaign http://www.bjcancer.com

**Keywords**: paclitaxel; oral administration; Cremophor EL

Paclitaxel is an important anticancer agent widely applied in the treatment of breast, ovarian and lung cancer and AIDS-related Kaposi's sarcoma (Huizing et al, 1995a; Rowinsky and Donehower, 1995). The drug is marketed as an intravenous (i.v.) formulation consisting of 6 mg ml$^{-1}$ paclitaxel dissolved in Cremophor EL:ethanol 1:1 v/v. Many different dosages and time schedules have been tested and further optimization of the clinical application is currently pursued. Recently, we reported about the oral route for administering paclitaxel to patients using the i.v. formulation as a drinking solution diluted with water (Meerum Terwogt et al, 1998, 1999). This work was based on preclinical studies highlighting the important role of P-glycoprotein (P-gp) in the oral bioavailability of paclitaxel (Sparreboom et al, 1997; Van Asperen et al, 1997). P-gp in the gut builds a barrier to many substrate xenotoxins and drugs, including paclitaxel. In patients, administration of 60 mg m$^{-2}$ paclitaxel with 15 mg kg$^{-1}$ cyclosporin A(CsA), a competitive inhibitor of both P-gp and cytochrome P450 3A4, significantly increased the oral bioavailability of paclitaxel by at least 7-fold and plasma concentrations rose from negligible to potentially therapeutic levels (Meerum Terwogt et al, 1998, 1999). To further enhance the systemic exposure to paclitaxel we performed a dose-escalation study of oral paclitaxel in combination with CsA (Malingré et al, 2000a). Although dose-escalation of oral paclitaxel from 60 to 300 mg m$^{-2}$ resulted in a significantly higher systemic exposure to paclitaxel, this increase was moderate and not proportional with dose. Similar results have been obtained by Rowinsky and co-workers (Britten et al, 2000). A mass balance study, which was performed in patients receiving the highest dose level (300 mg m$^{-2}$), revealed that a high fraction of the dose was recovered in the faeces as unchanged drug suggesting incomplete absorption (Malingré et al, 2000b). Moreover, high amounts of the co-solvent Cremophor EL were also recovered in faeces and the fractions of the dose of Cremophor EL and paclitaxel excreted in faeces were significantly correlated (Malingré et al, 2000b). We, therefore hypothesized that Cremophor EL limits the absorption of paclitaxel by entrapment of the drug in the gastro-intestinal tract.

This hypothesis was recently substantiated in preclinical models using mdr1ab P-gp knock-out mice (Bardelmeijer et al, 2000). Cremophor EL given at dosages relevant to cancer patients resulted in considerably decreased paclitaxel plasma levels and substantially increased faecal excretion of unchanged paclitaxel. Based on these preclinical results, we initiated this clinical study in which each patient received 60 mg m$^{-2}$ of oral paclitaxel, in combination with 15 mg kg$^{-1}$ of CsA, formulated in Cremophor EL (5 ml m$^{-2}$ or 15 ml m$^{-2}$) at one occasion and polysorbate 80 at the other with pharmacokinetic monitoring at both occasions.

*Received 20 February 2001*
*Revised 20 July 2001*
*Accepted 30 July 2001*

*Correspondence to:* MM Malingré

### PATIENTS AND METHODS

### Patient population

Patients with a histologically confirmed cancer refractory to current therapies were eligible for the study. Previous radiotherapy or chemotherapy was allowed, provided that the last treatment was at least 4 weeks prior to study entry and any resulting toxicities were resolved. Eligibility criteria included acceptable bone marrow (white blood cells > 3.0 × $10^9$ $l^{-1}$; platelets > 100 × $10^9$ $l^{-1}$), liver function (serum bilirubin ≤ 25 μmol $l^{-1}$; serum albumin ≥ 25 g $l^{-1}$, renal function (serum creatinine ≤160 μmol $l^{-1}$ or clearance ≥ 50 ml $min^{-1}$) and a World Health Organization (WHO) performance status ≤ 2. Patients were not eligible if they suffered from uncontrolled infectious disease, neurologic disease, bowel obstruction or symptomatic brain metastases. Other exclusion criteria were concomitant use of known P-gp inhibitors and chronic use of $H_2$-receptor antagonists or proton pump inhibitors. The study protocol was approved by the Medical Ethics Committee of the Institute and all patients gave written informed consent.

### Study design

6 patients received at 2 occasions, which were one week apart and randomized, oral paclitaxel at a dose of 60 mg $m^{-2}$ formulated in Cremophor EL/ethanol and the same oral paclitaxel dose formulated in polysorbate 80/ethanol. 2 cohorts of each 3 patients were made, one of which received Cremophor EL 5 ml $m^{-2}$ in the oral paclitaxel formulation and the other Cremophor EL 15 ml $m^{-2}$. The polysorbate 80 formulation was the same between the 2 cohorts. Prior to oral paclitaxel intake patients received 15 mg $kg^{-1}$ oral CsA.

For the oral paclitaxel formulation with Cremophor EL (5 and 15 ml $m^{-2}$), the standard i.v. formulation of paclitaxel was used (Taxol®; 6 mg $ml^{-1}$ paclitaxel in Cremophor EL: ethanol 1:1 v/v). 3 of the 6 patients received additional Cremophor EL (BASF, Brussels, Belgium) of 10 ml $m^{-2}$ to this formulation. The polysorbate 80 formulation was made similar to the i.v. Cremophor EL formulation of paclitaxel with replacement of Cremophor EL by polysorbate 80 (6 mg $ml^{-1}$ paclitaxel in polysorbate 80:ethanol 1:1 v/v). To all formulations 25 ml of water was added to decrease viscosity. Paclitaxel was retrieved from Hauser, Inc (Boulder, USA), polysorbate 80 from Kolb (Hedingen, Switzerland). CsA was administered as capsules (Neoral® Novartis, Basel, Switzerland; base: corn oil, polyoxyl 40 hydrogenated castor oil) 30 minutes prior to oral intake of paclitaxel.

An oral paclitaxel dose of 60 mg $m^{-2}$ was chosen for safety reasons. As we expected that the oral bioavailability of paclitaxel formulated in polysorbate 80 would approach the bioavailability of orally administered docetaxel, i.e. 90% (Malingré et al, 2001), a dose of 60 mg $m^{-2}$ oral paclitaxel administered within a time period of 2 weeks was considered to be therapeutic and safe. To prevent nausea and vomiting patients received 1 mg oral granisetron (Kytril®) approximately 2 hours prior to oral paclitaxel administration. In addition, patients received a light standard breakfast (2 crackers and a cup of tea) at least 2 hours prior to oral drug administration. Intake of food was not allowed until 2 hours following intake of paclitaxel.

2 weeks after the second oral course of paclitaxel, patients received i.v. paclitaxel (Taxol®) administered as a 3-hour infusion at a dose of 175 mg $m^{-2}$. If it was considered to be in their best interest patients continued on a 3-weekly schedule of i.v. paclitaxel. At the i.v. occasions, patients were premedicated to prevent hypersensitivity reactions with dexamethasone 20 mg orally 12 and 6 hours prior to, clemastine 2 mg i.v. 30 minutes prior to and cimetidine 300 mg i.v. shortly prior to paclitaxel administration. Oral doses were given without this premedication regimen as previous studies of oral paclitaxel have revealed that the co-solvent Cremophor EL, suspect of causing the hypersensitivity reactions (Dye and Watkins, 1980), was not absorbed following oral administration of paclitaxel (Meerum Terwogt et al, 1998, 1999; Malingré et al, 2000a).

### Sample collection

Blood samples for pharmacokinetic analyses were collected during the 2 oral courses. Samples were obtained in heparinized tubes pre-dosing, at 15, 30, 45, 60, 75 and 90 minutes and 2, 3, 4, 7, 10, 24 and 30 hours after paclitaxel ingestion. For the analysis of paclitaxel, blood samples were centrifuged, plasma was separated and immediately stored at –20˚C until analysis. For CsA analysis, 1 ml of whole blood was transferred, stored at 4˚C and analysed within one week after treatment. Urine was collected from 0–24 h and 24–30 h after paclitaxel administration. Samples were stabilized with a mixture of 5% Cremophor EL/ethanol 1:1 v/v to prevent paclitaxel precipitation and these samples were stored at –20˚C until analysis. The stools were collected in separate portions up to 6 days after dosing. The stools collected up to 30 hours after dosing were immediately stored at –20˚C, the stools collected from 30 hours up to 6 days after dosing were stored at the patients home and were frozen at day 6 at –20˚C. After defrosting, the faecal samples were homogenized in 10 parts of water, with a maximum of 2000 ml, and aliquots of the suspension were stored at –20˚C until analysis.

### Sample analysis

Paclitaxel and metabolite concentrations in plasma, urine and faeces were determined using validated high-performance liquid chromatography (HPLC) assays (Huizing et al, 1995b, 1995c; Sparreboom et al, 1995). All assays used 2′-methylpaclitaxel as the internal standard. Pretreatment of the plasma samples involved solid phase extraction (SPE) on Cyano Bond Elut columns. Pretreatment of urine samples involved liquid–liquid extraction (LLE) with *n*-butylchloride. Faecal samples were pretreated by LLE with diethyl ether followed by automated SPE using Cyano Bond Elut columns. The lower limit of quantitation for paclitaxel and metabolites was 10 ng ml for plasma, 25 ng ml for urine and 250 ng ml for faeces homogenates. CsA whole blood concentrations were analysed using a specific fluorescence polarization immunoassay (FPIA, Abbott Laboratories, Amstelveen, The Netherlands) (Chan et al, 1992). The concentration of Cremophor EL in faeces was measured using a validated HPLC assay (Sparreboom et al, 1996) with minor modifications and corrections for the presence of liberated free ricinoleic acid (Van Tellingen et al, 1999a).

### Pharmacokinetic analysis

Non-compartmental pharmacokinetic methods were applied to process the results (Gibaldi and Perrier, 1982). The maximal drug

*© 2001 Cancer Research Campaign* 

concentration ($C_{max}$) and time to maximal drug concentration ($T_{max}$) were obtained directly from the experimental data. The area under the concentration–time curve (AUC) was calculated by the trapezoidal rule up to the last measured time point (AUCt) with extrapolation to infinity using the terminal rate constant k. The excretion of paclitaxel, metabolites and Cremophor EL in faeces and urine was calculated relative to the administered dose. Statistical analysis of the data was performed using the non-parametric Wilcoxon matched-pairs signed-rank test. The a priori level of significance was $P = 0.05$.

### RESULTS

Formulation in polysorbate 80 resulted in significantly higher $C_{max}$ and AUC values of paclitaxel in comparison with the Cremophor EL formulations ($n = 6$; $P = 0.046$ for both parameters) (Figures 1, 2, Table 1). Compared to the 5 ml m$^{-2}$ Cremophor EL formulation, mean intrapatient differences in $C_{max}$ and AUC values were 1.5-fold, whereas these differences were 3.9-, and 3.2-fold, respectively with 15 ml m$^{-2}$ Cremophor EL. $T_{max}$ values of paclitaxel were significantly lower with the polysorbate 80 formulation compared to the Cremophor EL formulations ($n = 6$; $P = 0.046$).

Formulation of paclitaxel in polysorbate 80 also resulted in significantly higher $C_{max}$ and AUC values of CsA when compared to the Cremophor EL formulations ($n = 6$; $P = 0.028$ for both parameters) (Figure 3, Table 2). Compared to 5 ml m$^{-2}$ Cremophor EL, $C_{max}$ and AUC values of CsA were 1.4- and 1.3-fold higher, whereas these differences were 1.5- and 1.4-fold, respectively with 15 ml m$^{-2}$ Cremophor EL. $T_{max}$ values of CsA were not significantly different between the 2 paclitaxel formulations.

Excretion of unchanged paclitaxel in faeces was lower in the case of paclitaxel being formulated in polysorbate 80 compared to Cremophor EL (Figure 4, Table 3). However, these differences did not reach statistical significance ($n = 6$; $P = 0.115$). Importantly, in 2 patients receiving the Cremophor EL formulation, faeces collection was incomplete, resulting in relative low amounts of paclitaxel excreted in faeces. Excretion of the metabolite 6α-hydroxypaclitaxel was significantly higher with the polysorbate 80 formulation ($n = 6$; $P = 0.046$). Excretion of the metabolites 3′ p-hydroxypaclitaxel and 6α, 3′p-dihydroxypaclitaxel was not different between the 2 formulations. The amount of Cremophor EL excreted in faeces was 10.3 ± 4.9% of the administered dose for

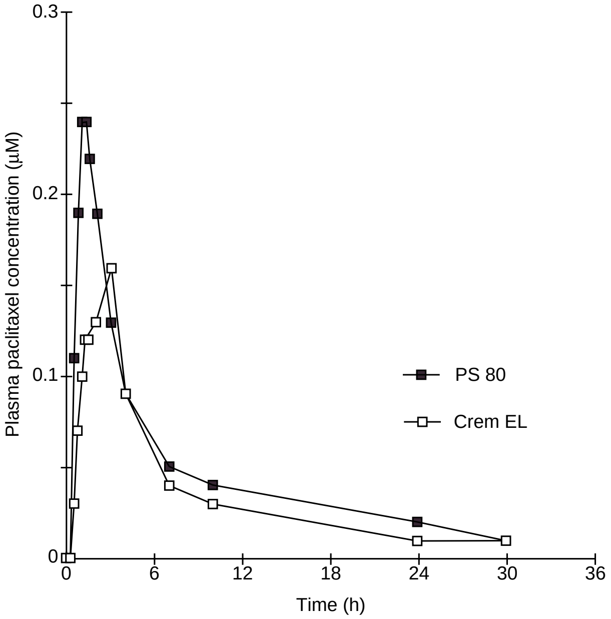

**Figure 2** Individual paclitaxel plasma concentration–time curves of a patient receiving oral paclitaxel formulated in Cremophor EL (Crem EL) 5 ml m$^{-2}$ and polysorbate 80 (PS 80)

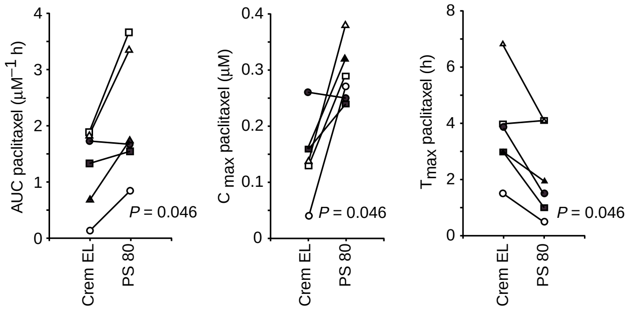

**Figure 1** Pharmacokinetic parameters of oral paclitaxel formulated in Cremophor EL (Crem EL) 5 ml m$^{-2}$ (closed symbols) or 15 ml m$^{-2}$ (open symbols) and polysorbate 80 (PS 80)

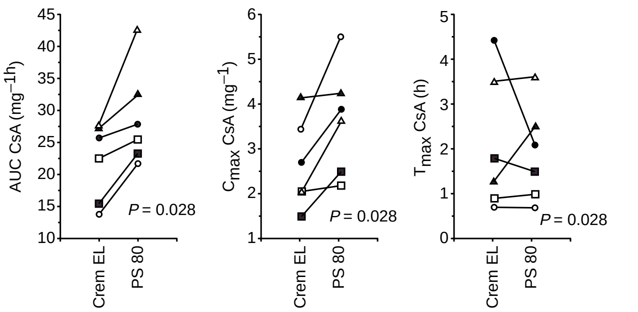

**Figure 3** Pharmacokinetic parameters of oral cyclosporin A (CsA) administered just prior to oral paclitaxel formulated in Cremophor EL (Crem EL) 5 ml m$^{-2}$ (closed symbols) or 15 ml m$^{-2}$ (open symbols) and polysorbate 80 (PS 80)

**Table 1** Pharmacokinetic parameters of oral paclitaxel (60mg m$^{-2}$) formulated in polysorbate 80 (PS 80) and Cremophor EL (Crem EL) 5 ml m$^{-2}$ (cohort 1) or 15 ml m$^{-2}$ (cohort 2). Data are presented as means ± SD

| cohort | AUC (μM h$^{-1}$) | | $C_{max}$ (μM) | | $T_{max}$ (h) | |
|---|---|---|---|---|---|---|
| | Crem EL | PS 80 | Crem EL | PS 80 | Crem EL | PS 80 |
| 1 | 1.25 | 1.66 | 0.19 | 0.27 | 3.3 | 1.5 |
| | (0.52) | (0.11) | (0.06) | (0.04) | (0.5) | (0.5) |
| 2 | 1.29 | 2.61 | 0.10 | 0.31 | 4.1 | 2.9 |
| | (0.99) | (1.54) | (0.06) | (0.06) | (2.7) | (2.1) |

*© 2001 Cancer Research Campaign*

**Table 2** Pharmacokinetic parameters of oral cyclosporin A (15 mg $kg^{-1}$) administered just prior to oral paclitaxel (60 mg $m^{-2}$) formulated in polysorbate 80 (PS 80) and Cremophor EL (Crem EL) 5 ml $m^{-2}$ (cohort 1) or 15 ml $m^{-2}$ (cohort 2). Data are presented as means ± SD

| Cohort | AUC (mg $l^{-1}$h) | | $C_{max}$ (mg $l^{-1}$) | | $T_{max}$ (h) | |
|---|---|---|---|---|---|---|
| | Crem EL | PS 80 | Crem EL | PS 80 | Crem EL | PS 80 |
| 1 | 22.9 | 27.9 | 2.79 | 3.54 | 2.5 | 2.0 |
| | (6.5) | (4.6) | (1.34) | (0.93) | (1.7) | (0.5) |
| 2 | 21.4 | 29.9 | 2.53 | 3.78 | 1.7 | 1.8 |
| | (7.0) | (11.1) | (0.80) | (1.65) | (1.6) | (1.6) |

**Table 3** Faecal excretion of paclitaxel and the metabolites 6α-hydroxypaclitaxel (6α-HP), 3′p-hydroxypaclitaxel (3′p-HP) and 6α,3′p-dihydroxypaclitaxel (6α,3′p-DHP) after oral paclitaxel administration (60 mg $m^{-2}$) formulated in polysorbate 80 (PS 80) and Cremophor EL (Crem EL) 5 ml $m^{-2}$ (cohort 1) or 15 ml $m^{-2}$ (cohort 2). Data are presented as means ± SD

| Cohort | Paclitaxel (% of dose) | | 6α-HP (% of dose) | | 3′p-HP (% of dose) | | 6α,3′p-DHP (% of dose) | | total recovery (% of dose) | |
|---|---|---|---|---|---|---|---|---|---|---|
| | Crem EL | PS 80 | Crem EL | PS 80 | Crem EL | PS 80 | Crem EL | PS 80 | Crem EL | PS 80 |
| 1 | 25.9 | 24.4 | 22.5 | 31.4 | 2.3 | 2.5 | 4.3 | 6.8 | 55.0 | 65.1 |
| | (2.5) | (10.0) | (4.8) | (9.1) | (1.4) | (1.8) | (2.9) | (3.9) | (7.0) | (6.2) |
| 2 | 38.8 | 18.3 | 22.3 | 23.7 | 2.8 | 1.7 | 2.5 | 2.5 | 66.4 | 46.2 |
| | (13.0) | (15.5) | (12.6) | (13.7) | (0.3) | (0.5) | (0.7) | (0.7) | (3.0) | (2.8) |

the 5 ml $m^{-2}$ group and 20.9 ± 16.0% of the administered dose for the 15 ml $m^{-2}$ group. The total fraction of Cremophor EL excreted in faeces was significantly correlated with the amount of paclitaxel excreted in faeces ($P$ = 0.019, r = 0.886) ($n$ = 6) (Figure 5).

Excretion of orally administered paclitaxel in urine was minimal, as observed previously (Malingré et al, 2000a, 2000b). Urinary excretion of paclitaxel was 2.4 ± 1.1% of the administered dose for the polysorbate 80 formulation ($n$ = 6), 2.1 ± 0.7% of the administered dose for the 5 ml $m^{-2}$ Cremophor EL formulation ($n$ = 3) and 2.6 ± 0.7% of the administered dose for the 15 ml $m^{-2}$ Cremophor EL formulation ($n$ = 2). Urine collection of one patient was incomplete due to loss of urine during collection.

### DISCUSSION

The results of this study show that the presence of Cremophor EL in the i.v. formulation of paclitaxel used orally reduces the absorption of paclitaxel from the gut. Formulation of paclitaxel in polysorbate 80 resulted in a significant increase in the $C_{max}$ and AUC values of paclitaxel. The excretion of unchanged paclitaxel in faeces was substantially lower for the polysorbate 80 formulation and indicates an improved oral uptake. At the same time, excretion of the major paclitaxel metabolite 6α-hydroxypaclitaxel was significantly increased, which is also indicative of increased absorption of the drug. The relationship between reduction in the absorption of paclitaxel and Cremophor EL was evident. The amount of paclitaxel excreted in faeces was significantly

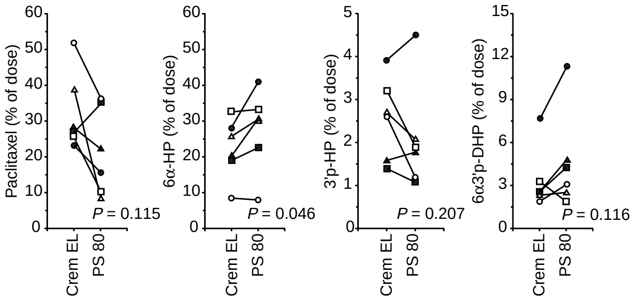

**Figure 4** Faecal excretion of paclitaxel and the metabolites 6α-hydroxypaclitaxel (6α-HP), 3′p-hydroxypaclitaxel (3′p-HP) and 6α,3′p-dihydroxypaclitaxel (6α,3′p-DHP) after oral paclitaxel administration in Cremophor EL (Crem EL) 5 ml $m^{-2}$ (closed symbols) or 15 ml $m^{-2}$ (open symbols) and polysorbate 80 (PS 80)

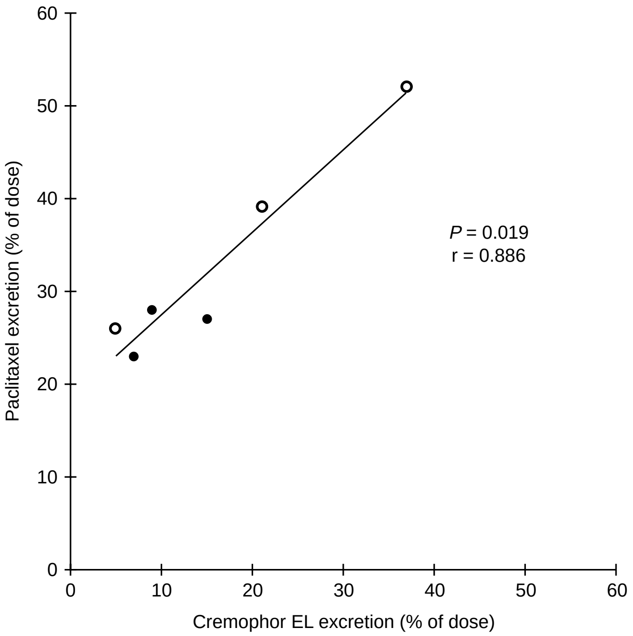

**Figure 5** Faecal excretion of paclitaxel versus the faecal excretion of Cremophor EL after oral paclitaxel administration in Cremophor EL 5 ml $m^{-2}$ (closed symbols) of 15 ml $m^{-2}$ (open symbols)

correlated with the amount of Cremophor EL excreted in faeces. Interestingly, Cremophor EL of the paclitaxel formulation also reduced the absorption of CsA. Significantly higher $C_{max}$ and AUC values of CsA were observed when paclitaxel was formulated in polysorbate 80 rather than in Cremophor EL. This result is in line with those obtained in our previously performed dose-escalation study of oral paclitaxel given together with a constant dose of CsA (Malingré et al, 2000a). Apparently, absorption of orally administered paclitaxel and CsA are influenced by Cremophor EL by the same mechanism.

The results of this clinical study are in good agreement with our preclinical data (Bardelmeijer et al, 2000). In mdr1ab P-gp knock-out mice, receiving 10 mg $kg^{-1}$ paclitaxel in the standard formulation, only about 7% of the dose was excreted in faeces as unchanged drug, suggesting almost complete absorption from the gastro-intestinal tract. However, when the dose of Cremophor EL was increased by 7-fold, faecal excretion of unchanged drug increased to 35% of the dose. Moreover, the plasma $C_{max}$ and AUC values of paclitaxel were 4- and 1.6-fold lower, respectively. For reasons of availability our preclinical and clinical studies with oral paclitaxel performed thus far have used the standard i.v. formulation of paclitaxel. Cremophor EL, a mixture of polyoxyethylated triglycerides, is an essential compound in this formulation used to solubilize paclitaxel in aqueous dilutions by formation of micelles, which include the drug molecules within their hydrophobic core. After oral paclitaxel administration, Cremophor EL was assumed to be degraded in the gastro-intestinal tract as paclitaxel and Cremophor EL levels recovered in faeces of mdrla P-gp knock-out mice were very low (Sparreboom et al, 1997; Bardelmeijer et al, 2000). However, in our clinical study of oral paclitaxel 300 mg $m^{-2}$, a substantial fraction of the dose of Cremophor EL, i.e. 32%, was recovered in faeces together with 61% of the dose of paclitaxel, indicative for incomplete degradation of Cremophor EL and poor uptake of paclitaxel (Malingré et al, 2000b). By use of an in vitro assay we have shown that micelles are being formed in the intestines of mice at Cremophor EL concentrations of 0.33% w/v and higher (Bardelmeijer et al, 2000). With the addition of extra Cremophor EL to mdrlab P-gp knock-out mice, the levels of Cremophor EL in the intestinal contents were approximately 10-fold higher. It could then be concluded that the mechanism of interaction between paclitaxel and Cremophor EL rests on the property of Cremophor EL to form micelles, which entrap paclitaxel thus reducing the availability of paclitaxel for uptake (Bardelmeijer et al, 2000). In line with this hypothesis it is likely that the oral bioavailability of CsA is similarly affected.

The selection of polysorbate 80 as vehicle used to replace Cremophor EL was based on the following considerations (1) the very good oral bioavailability of docetaxel, a taxane drug formulated in polysorbate 80/ethanol (Malingré et al, 2001) and (2) the rapid degradation of polysorbate 80 by esterases in plasma (Van Telligen et al, 1999b). Initially, we planned to formulate paclitaxel in polysorbate 80 similar to docetaxel (Taxotere®) with 20 mg drug per 0.5 ml polysorbate 80 and 1.5 ml ethanol 13% g $g^{-1}$. However, no clear solution of paclitaxel could be made. Therefore, it was decided to formulate paclitaxel in polysorbate 80 similar to the i.v. Cremophor EL formulation with 6 mg drug per 0.5 ml polysorbate 80 and 0.5 ml absolute ethanol. This formulation was clear and appeared feasible. Paclitaxel formulated in polysorbate 80 resulted in a mean paclitaxel excretion in faeces of 21%, which suggests incomplete uptake of the drug from the gut. We previously determined that after i.v. administration of paclitaxel (175 mg $m^{-2}$) only 9% of the drug was recovered as unchanged paclitaxel in faeces (Malingré et al, 2000b). The incomplete uptake of oral paclitaxel formulated in polysorbate 80 may be caused by the relatively high amount of polysorbate 80, which may have, to a certain extent, similar capabilities as Cremophor EL of forming micelles, especially at higher concentrations. We are currently investigating a new formulation of oral paclitaxel with a different solvent, which may shed more light on this issue.

In conclusion, the results show that the co-solvent Cremophor EL is an important factor limiting the absorption of orally administered paclitaxel from the intestinal lumen, in particular at the higher dose levels. Development of a better, non-Cremophor EL-based drug formulation is needed in order to increase the usefulness of the oral route of paclitaxel.

### ACKNOWLEDGEMENTS

This work was supported by the Dutch Cancer Society (NKI project 98–1799).

### REFERENCES

Bardelmeijer HA, Ouwehand M, Malingré M, Schellens JHM, Beijnen JH and Van Tellingen O (2000) Entrapment by Cremophor EL decreases the absorption of paclitaxel from the gut. *Clin Cancer Res* **6**: (Suppl 11), abstract 189

Britten CD, Baker SD, Denis LJ, Johnson T, Drengler R, Siu LL, Duchin K, Kuhn J and Rowinsky EK (2000) Oral paclitaxel and concurrent cyclosporin A: Targeting clinically relevant systemic exposure to paclitaxel. *Clin Cancer Res* **6**: 3459–3468

Chan GL, Weinstein SS, Lefor WW, Spoto E, Kahana L and Shires DL (1992) Relative performance of specific and nonspecific fluorescence immunoassay for cyclosporin in transplant patients. *Ther Drug Monit* **14**: 42–47

Dye D and Watkins J (1980) Suspected anaphylactic reaction to Cremophor EL. *BMJ* **280**: 1353

Gibaldi M and Perrier D (1982) Noncompartmental analysis based on statistical moment theory. In: *Pharmacokinetics*, Gibaldi M and Perrier D (eds), pp 409–417. Marcel Dekker: New York

Huizing MT, Sewberath Misser VH, Pieters RC, Ten Bokkel Huinink WW, Veenhof CHN, Vermorken JB, Pinedo HM and Beijnen JH (1995a) Taxanes: a new class of antitumor agents. *Cancer Invest* **13**: 381–404

Huizing MT, Sparreboom A, Rosing H, Van Tellingen O, Pinedo HM and Beijnen JH (1995b) Quantification of paclitaxel metabolites in human plasma by high-performance liquid chromatography. *J Chromatogr B* **674**: 261–268

Huizing MT, Rosing H, Koopman FJ, Keung ACF, Pinedo HM and Beijnen JH (1995c) High-performance liquid chromatographic procedures for the quantitative determination of paclitaxel (Taxol) in human urine. *J Chromatogr B* **664**: 373–382

Malingré MM, Meerum Terwogt JM, Beijnen JH, Rosing H, Koopman FJ, Van Tellingen O, Duchin K, Ten Bokkel Huinink WW, Swart M, Lieverst J and Schellens JHM (2000a) Phase I and pharmacokinetic study of oral paclitaxel. *J Clin Oncol* **18**: 2468–2475

Malingré M, Schellens JHM, Van Tellingen O, Rosing H, Koopman FJ, Duchin K, Ten Bokkel Huinink WW, Swart M and Beijnen JH (2000b) Metabolism and excretion of paclitaxel after oral administration in combination with cyclosporin A and after i.v. administration. *Anti-Cancer Drugs* **11**: 813–820

Malingré MM, Richel DJ, Beijnen JH, Rosing H, Koopman FJ, Ten Bokkel Huinink WW, Schot ME and Schellens JHM (2001) Co-administration of cyclosporin A strongly enhances the oral bioavailability of docetaxel. *J Clin Oncol* **19**: 1160–1166

Meerum Terwogt JM, Beijnen JH, Ten Bokkel Huinink WW, Rosing H and Schellens JHM (1998) Co-administration of cyclosporin enables oral therapy with paclitaxel. *Lancet* **352**: 285

Meerum Terwogt JM, Malingré MM, Beijnen JH, Ten Bokkel Huinink WW, Rosing H, Koopman FJ, Van Tellingen O, Swart M and Schellens JHM (1999) Co-administration of cyclosporin A enables oral therapy with paclitaxel. *Clin Cancer Res* **5**: 3379–3384

Rowinsky EK and Donehower RC (1995) Paclitaxel (Taxol). *N Engl J Med* **332**: 1004–1014

 *© 2001 Cancer Research Campaign*

Sparreboom A, Van Tellingen O, Nooijen WJ and Beijnen JH (1995) Determination of paclitaxel and metabolites in mouse plasma, tissues, urine and feces by semi-automated reversed-phase high-performance liquid chromatography. *J Chromatogr B* **664**: 383–391

Sparreboom A, Van Tellingen O, Huizing MT, Nooijen WJ and Beijnen JH (1996) Determination of Polyoxyethyleneglycerol Triricinoleate 35 (Cremophor EL) in plasma by pre-column derivatization and reversed-phase high-performance liquid chromatography. *J Chromatogr B* **681**: 355–362

Sparreboom A, Van Asperen J, Mayer U, Schinkel AH, Smit JW, Meijer DKF, Borst P, Nooijen WJ, Beijnen JH and Van Tellingen O (1997) Limited oral bioavailability and active epithelial secretion of paclitaxel caused by P-glycoprotein in the intestine. *Proc Natl Acad Sci USA* **94**: 2031–2035

Van Asperen J, Van Tellingen O, Sparreboom A, Schinkel AH, Borst P, Nooijen WJ and Beijnen JH (1997) Enhanced oral bioavailability of paclitaxel in mice treated with the P-glycoprotein blocker SDZ PSC 833. *Br J Cancer* **76**: 1181–1183

Van Tellingen O, Huizing MT, Nannan Panday VR, Schellens JHM, Nooijen WJ and Beijnen JH (1999a) Cremophor EL causes (pseudo) nonlinear pharmacokinetics of paclitaxel in patients. *Br J Cancer* **81**: 330–335

Van Tellingen O, Beijnen JH, Verweij J, Scherrenburg EJ, Nooijen WJ and Sparreboom A (1999b) Rapid esterase-sensitive breakdown of polysorbate 80 and its impact on the plasma pharmacokinetics of docetaxel and metabolites in mice. *Clin Cancer Res* **5**: 2918–2923

*© 2001 Cancer Research Campaign* 
